# Mevalonate kinase represses anthocyanin biosynthesis via sucrose transporters and gibberellin synthesis pathways in arabidopsis

**DOI:** 10.1007/s00299-026-03883-w

**Published:** 2026-06-15

**Authors:** Jinku Kang, Sua Cho, Kiyoon Kang, Daewon Kim, Sang-Il Bae, Eunji Shin, So-Yon Park, Gary Stacey, Nam-Chon Paek, Sung-Hwan Cho

**Affiliations:** 1 Department of Agriculture, Forestry and Bioresources, Research Institute of Agriculture and Life Sciences, Seoul National University, Seoul, Republic of Korea; 2 Plant Genomics and Breeding Institute, Seoul National University, Seoul, Republic of Korea; 3 Division of Life Sciences, Incheon National University, Incheon, Republic of Korea; 4 Division of Life Science and Division of Applied Life Science, Plant Molecular Biology and Biotechnology Research Center, Gyeongsang National University, Jinju, Republic of Korea; 5 Division of Plant Science and Biotechnology, University of Missouri, Columbia, MO USA; 6 Department of Environmental and Biological Chemistry, Chungbuk National University, Cheongju, Republic of Korea

**Keywords:** Anthocyanin, Arabidopsis, Gibberellic acid, Mevalonate pathway, *MVK*, *SUC1*

## Abstract

**Key message:**

Arabidopsis MVK negatively regulates sucrose-induced anthocyanin accumulation by modulating SUC1-mediated sucrose transport and gibberellin homeostasis.

**Abstract:**

Anthocyanins are flavonoid pigments that function as crucial modulators of plant responses to environmental stressors by mitigating oxidative damage and facilitating cellular adaptation. Anthocyanin biosynthesis is tightly regulated by transcriptional networks that respond to developmental cues and external stimuli. In this study, we identify mevalonate kinase (MVK), a critical enzyme in the cytosolic isoprenoid biosynthesis pathway, as a repressor of sucrose-induced anthocyanin production in arabidopsis. Loss-of-function *mvk-1* mutants show increased anthocyanin levels compared to wild-type (WT) plants under high-sucrose conditions. The expression of anthocyanin biosynthetic and regulatory genes, such as *CHS*, *DFR*, and *MYB75*/*PAP1*, increases in *mvk-1* mutants grown in the presence of high sucrose, and *mvk-1* mutants exhibit elevated sucrose accumulation through the upregulation of sucrose transporters compared to WT under high-sucrose conditions. Furthermore, reduced gibberellic acid (GA) in *mvk-1* mutants resulted in stabilization of repressors of GA signaling, known as DELLA proteins, thereby facilitating sucrose-induced anthocyanin accumulation. Our findings suggest that MVK negatively regulates sucrose-induced anthocyanin biosynthesis by modulating sucrose transport and GA homeostasis in arabidopsis.

**Supplementary Information:**

The online version contains supplementary material available at 10.1007/s00299-026-03883-w.

## Introduction

Anthocyanins, a pivotal class of water-soluble flavonoids, are responsible for the pigmentation of both vegetative (leaves, stems, and roots) and reproductive (flowers and fruits) organs in plants. These pigments are not only esthetic, but also serve crucial ecological functions, such as attracting pollinators and seed dispersers, which are essential for reproduction and survival (Harborne and Williams 2000). Beyond their ecological roles, anthocyanins are widely recognized for their role in abiotic stress responses, particularly through their antioxidant properties that mitigate the oxidative damage caused by reactive oxygen species (ROS) (Grotewold 2006; Xu et al. 2017). Additionally, anthocyanins offer nutritional benefits to humans, making anthocyanin-rich plants increasingly important research targets (Khoo et al. 2017).

The biosynthesis of anthocyanins is regulated by a combination of developmental cues and environmental signals, including light, temperature, and several endogenous molecules (LaFountain and Yuan 2021). Sucrose, for example, is known to promote anthocyanin biosynthesis (Solfanelli et al. 2006). In addition, phytohormones, including ethylene, jasmonic acid (JA), gibberellic acid (GA), abscisic acid (ABA), and cytokinin (CK), interact with sucrose signaling pathways, collectively modulating anthocyanin production (Das et al. 2012; Loreti et al. 2008).

Anthocyanins are synthesized in the cytosol, then modified into various derivatives, and then subsequently transported into vacuoles. In arabidopsis (*Arabidopsis thaliana*), structural genes involved in anthocyanin biosynthesis are classified as early (EBGs) or late (LBGs) biosynthetic genes (Deroles 2008). The pathway starts with the condensation of one molecule of 4-coumaroyl-CoA and three molecules of malonyl-CoA, which leads to the formation of naringenin chalcone. EBGs, including chalcone synthase (CHS), chalcone isomerase (CHI), flavonol 3-hydroxylase (F3H), and flavonol 3-hydroxylase (F3′H) further metabolize naringenin chalcone, leading to the production of flavonols. Subsequently, LBGs, including dihydroflavonol-4-reductase (DFR), leucoanthocyanidin dioxygenase (LDOX), anthocyanidin reductase (ANR), and UDP-Glc:flavonoid 3-O-glucosyltransferase (UF3GT), facilitate the final steps that produce anthocyanins and proanthocyanidins (Holton and Cornish 1995).

Anthocyanin biosynthesis is tightly regulated at the transcriptional level by the MBW (MYB-bHLH-WD40) complex, which consists of R2R3-MYB, bHLH, and WD40-repeat proteins (Hichri et al. 2011). In arabidopsis, the MBW complex includes the R2R3-MYB proteins (MYB75/PAP1, MYB90/PAP2, MYB113, and MYB114), bHLH transcription factors [TRANSPARENT TESTA 8 (TT8), GLABRA 3 (GL3), and ENHANCER OF GLABRA 3 (EGL3)], and the WD40-repeat protein TRANSPARENT TESTA GLABRA 1 (TTG1) (Broun 2005; Gonzalez et al. 2008). This complex orchestrates anthocyanin biosynthesis by activating the expression of structural genes, such as *Phenylalanine Ammonia Lyase* (*PAL*), *CHS*, *CHI*, *F3H*, *DFR*, *ANS*, and *UF3GT*, ultimately promoting anthocyanin accumulation in vegetative and reproductive tissues (Das et al. 2012). Recent studies in arabidopsis revealed that GOLDEN2-LIKE 1 (GLK1) promotes anthocyanin accumulation by directly interacting with MYB75, MYB90, and MYB113, thereby boosting their transcriptional activity (Li et al. 2023). In addition, energy deficiency suppresses anthocyanin accumulation through the action of Sucrose Non-Fermenting-1-Related Protein Kinase1 (SnRK1), a master metabolic regulator. SnRK1 destabilizes MYB75, thus repressing MBW-mediated transcription and anthocyanin production under low-energy conditions (Broucke et al. 2023). Homologs, such as FvMYB10, FvMYB41, and FvMYB105, interact with bHLH partners, such as FvbHLH33 and FvMYC1, to regulate stage-specific anthocyanin and proanthocyanidin synthesis during woodland strawberry fruit ripening (Xu et al. 2021). Importantly, MBW activity is modulated by internal and external signals, such as hormones and sugars. DELLA proteins, which mostly act as repressors of GA signaling, promote anthocyanin biosynthesis by sequestering the JAZ and MYBL2 repressors. This enables MBW activation (Xie et al. 2016). In addition, sucrose enhances anthocyanin biosynthesis by stabilizing DELLA proteins and inducing the expression of MBW-regulated genes, such as *MYB75/PAP1*, *CHS*, and *DFR* (Li et al. 2014).

Given its dual role as both a carbon source and a signaling molecule, sucrose not only provides metabolic substrates but also functions as a key regulator that integrates hormonal and transcriptional networks to promote anthocyanin accumulation (Loreti et al. 2008; Sakr et al. 2018). Sucrose content in plants may increase due to alterations in sucrose metabolism or the activity of sucrose transporters (Julius et al. 2017). Sucrose transporters (SUCs) are sucrose-proton symporters involved in sucrose translocation (Braun 2022). Nine putative *SUC* genes have been identified in arabidopsis, and some of which are directly linked to anthocyanin accumulation. For example, SUC1 is essential for sucrose-induced anthocyanin accumulation; sucrose-induced anthocyanin accumulation is inhibited in *SUC1* knockout mutants (Sivitz et al. 2008). Additionally, the arabidopsis *pho3* mutants, which are defective in SUC2 function, exhibit enhanced anthocyanin accumulation (Lloyd and Zakhleniuk 2004). Among *SUC* genes, *SUC5* is specifically expressed in the endosperm; *SUC6* and *SUC7* encode aberrant proteins; and *SUC8* and *SUC9* are predominantly expressed in floral tissues (Baud et al. 2005; Meyer et al. 2000; Sauer et al. 2004).

The mevalonate (MVA) pathway functions in the cytosol of plant cells and plays a crucial role in primary metabolism by producing isoprenoids, sterols, and other key metabolites (Pulido et al. 2012). Specifically, the MVA pathway provides essential precursors for the synthesis of brassinosteroid (BR), ubiquinone, and dolichol. It also supplies farnesyl diphosphate (FPP) for protein prenylation, which is vital for intracellular signaling (Vranová et al. 2012). Furthermore, the MVA pathway significantly influences the homeostasis of other phytohormones, including GA, CK, and ABA, through metabolic crosstalk with the MEP pathway (Laule et al. 2003; Kasahara et al. 2002). The MEP pathway is conserved across plants, fungi, and animals, supporting diverse physiological processes (Miziorko 2011; Ruiz-Sola et al. 2016; Yang et al. 2021). Mevalonate kinase (MVK) is a key enzyme within this pathway that phosphorylates mevalonic acid to produce mevalonate 5-phosphate (Riou et al. 1994). In arabidopsis, *MVK* is broadly expressed, with relatively high expression in roots and reproductive tissues such as inflorescences (Lluch et al. 2000). A recent study showed that arabidopsis MVK is a direct phosphorylation target of P2K1, which activates the MVA pathway in response to extracellular ATP (eATP) elicitation (Cho et al. 2022). The relationship between the MVA pathway and anthocyanin production has been studied in apple trees (*Malus domestica* Borkh). This pathway produces isoprenoids and sterols, and it has been shown to influence anthocyanin accumulation by positively regulating IAA and ABA synthesis while inhibiting GA synthesis (Flores-Pérez et al. 2010; Li et al. 2018). However, the mechanism by which the MVA pathway regulates anthocyanin production in plants remains unclear.

In this study, we aimed to elucidate the role of MVK, a core enzyme of the cytosolic isoprenoid biosynthesis pathway, in the regulation of sucrose-induced anthocyanin biosynthesis in arabidopsis. While the MVA pathway has been associated with various metabolic and hormonal signaling events, its connection to anthocyanin production in response to sucrose is not well understood. Our findings reveal that *mvk-1* mutants accumulate more anthocyanins than the WT under high-sucrose conditions. This phenotype is accompanied by elevated expression of genes involved in anthocyanin biosynthesis, such as *CHS* and *DFR*, as well as of transcriptional regulators, such as *MYB75/PAP1*. Furthermore, we demonstrate that MVK negatively regulates anthocyanin accumulation through two distinct mechanisms. First, MVK inhibits *SUC1* expression. Genetic analysis of the *mvk-1 suc1-5* double mutants further revealed that this regulation is mediated by a SUC1-dependent pathway. Second, mutation of *MVK* reduces GA levels, thereby promoting stabilization of the DELLA protein. Together, our findings reveal an uncharacterized role for MVK as a negative regulator of sucrose-induced anthocyanin biosynthesis. This role integrates sugar transport and hormonal signaling into a coordinated regulatory network.

## Materials and methods

### Plant material and growth conditions

Wild-type aequorin-expressing transgenic arabidopsis ColQ (Col-0 background) plants were provided by Marc Knight (Knight et al. 1996). The *mvk-1* mutant (ColQ background) has been described previously (Cho et al. 2022). Arabidopsis seeds were surface-sterilized and sown on half-strength Murashige and Skoog (MS) medium supplemented with 0% (w/v) sucrose, 0.5% (w/v) agar (MB Gellan Gum, Cat. No. MB-G4367), and 0.05% (w/v) MES (pH 5.7). After 3 days of cold stratification at 4 °C, the plates were placed vertically in a growth chamber set to a 16-h light/8-h dark photoperiod at 22 °C with a light intensity of 100 μmol m⁻^2^ s⁻^1^.

### Generating CRISPR-Cas9

The *suc1-5* mutant was generated via CRISPR/Cas9-mediated genome editing. A single guide RNA (sgRNA, 5`-CTCGATCCCTGGGACATTCCTGG-3`) was designed to target the *SUC1* coding region using the CRISPR Direct program (http://crispr.dbcls.jp/) (Naito et al. 2015). The tRNA–gRNA–Cas9 fragment was inserted into the *pRGEB32* vector (Xie et al. 2015). This binary vector was transformed into the *Agrobacterium tumefaciens* strain GV3101, which was then used to transform arabidopsis plants via the floral dip method (Clough and Bent 1998). The homozygous lines were screened based on hygromycin resistance. This selection was confirmed by directly sequencing PCR-amplified genomic products with primers targeting the regions listed in Supplementary Table 1.

### Anthocyanin assay

Anthocyanins were extracted using a modified version of a previously described method (Nakata et al. 2013). Ten-day-old arabidopsis seedlings were transferred to half-strength MS medium supplemented with or without 3% sucrose (w/v) and grown for an additional three days. Rosette leaves were then extracted in a solution of 45% methanol and 5% acetic acid (v/v). The relative anthocyanin content was determined spectrophotometrically by measuring the absorbance at 520 and 657 nm. The relative values were then calculated.

### RNA extraction and RT-qPCR analysis

Total RNA was extracted from whole arabidopsis seedlings using GeneAll Hybrid-R (GeneAll Biotechnology, Republic of Korea) according to the manufacturer’s instructions. First-strand cDNA was synthesized from 2 μg of total RNA using M-MLV reverse transcriptase (Promega, Madison, WI). RT-qPCR was performed using GoTaq PCR Mix (Promega, Madison, USA) according to the manufacturer's instructions, and qPCR was performed using a LightCycler 2.0 system (Roche Diagnostics, Mannheim, Germany). Transcript levels were normalized to the expression of the *UBQ* gene. The primers used for RT-qPCR analysis are provided in Supplementary Table 1.

### Sucrose measurements

For sucrose extraction, 20 mg of arabidopsis rosette leaves (fresh weight) was ground in liquid nitrogen and extracted using 80% ethanol (v/v). The extracted samples were then centrifuged at 12,000 × *g* for 10 min at 4 °C. The supernatant was filtered before analysis. Soluble sucrose content was quantified by high-performance liquid chromatography (HPLC) using a Dionex Ultimate 3000 system (Thermo Fisher Scientific, Sunnyvale, CA) equipped with a Shodex RI-101 refractive index detector (Shodex, Tokyo, Japan) at the Seoul National University NICEM. Separation was performed on a Sugar-Pak column (Waters, 300 mm × 6.5 mm) at 70 °C. The mobile phase consisted of ultrapure water (Milli-Q grade) at a flow rate of 0.5 mL/min. The injection volume was set at 10 μL for each sample. Chromeleon ver. 6 software was used for data acquisition and processing. Calibration was carried out using a sucrose standard (Sigma, 99.5% purity). Quantification was performed by comparing the peak areas to the samples to the standard curve generated from the known sucrose concentrations.

### DELLA protein stability

For the DELLA protein stability analysis, five-day-old seedlings were grown in 6-well plates with 1 mL liquid half-strength MS medium (pH 5.7) supplemented with 0% sucrose (w/v) under long-day conditions (16 h light/8 h dark, 21 °C). After three days, 5% (w/v) sucrose was added for an additional three days. Then, the seedlings were treated with 10 μM GA for 2 h. Total protein was extracted using extraction buffer containing 50 mM Tris–HCl (pH 7.5), 250 mM NaCl, 10 mM MgCl_2_, 1 mM EDTA, 0.5% Triton X-100, 10% glycerol, 1 mM dithiothreitol (DTT), 0.2 mM phenylmethanesulfonyl fluoride (PMSF), and 1 × Pierce protease inhibitor (Thermo Fisher, Rockford, IL). The extracted proteins were mixed with 5 × Laemmli loading buffer containing 10% SDS, 50% glycerol, 0.01% bromophenol blue, 10% - ṣ mercaptoethanol, and 0.3 M Tris–HCl (pH 6.8), and boiled at 95 °C for 5 min. The total extracted proteins were separated by 10% SDS-PAGE gel electrophoresis, and proteins were transferred to a PVDF membrane (Immobilon^®^-P, Millipore) using a semi-dry transfer system (Trans-Blot^®^ SD, Bio-Rad, Hercules, CA). After blocking with 5% skim milk, the membrane was incubated with the RGA/DELLA antibody (Agrisera, Cat. No. AS11-1630, diluted 1:1000) in 5% skim milk for 2 h. The membrane was then washed three times and incubated with the secondary goat anti-rabbit HRP antibody (Santa Cruz, Cat. No. sc-2004, diluted 1:10,000) for 2 h. The membrane was subsequently washed five times in TBST (50 mM Tris, 150 mM NaCl, 0,05% Tween 20), incubated with the Pierce SuperSignal^®^ West Pico chemiluminescent substrate (Thermo Scientific, Cat. No. 34578) for 1 min and exposed to film.

### GA quantification

GA quantification was analyzed using the previously described method (Xin et al. 2020). Briefly, 1 g of 10-day-old whole seedlings was ground into a fine powder in liquid nitrogen, and the samples were freeze-dried for three days. One mL of a solution containing 80% methanol (v/v) was added to each sample, which was then incubated for 12 h at 4 °C. The samples were then centrifuged at 12,000 × *g* for 15 min at 4 °C. The solvent was then dried down using a SpeedVac concentrator at room temperature (25 °C). The dried pellets were resuspended in 100 μL of an 80% methanol (v/v) solution. The resuspended samples were immediately subjected to liquid chromatography–mass spectrometry (LC–MS) hormonal analysis at Seoul National University NICEM (Seoul, Republic of Korea).

## Results

### *mvk-1* mutants exhibit enhanced sucrose-induced anthocyanin accumulation

**Fig. 1:**
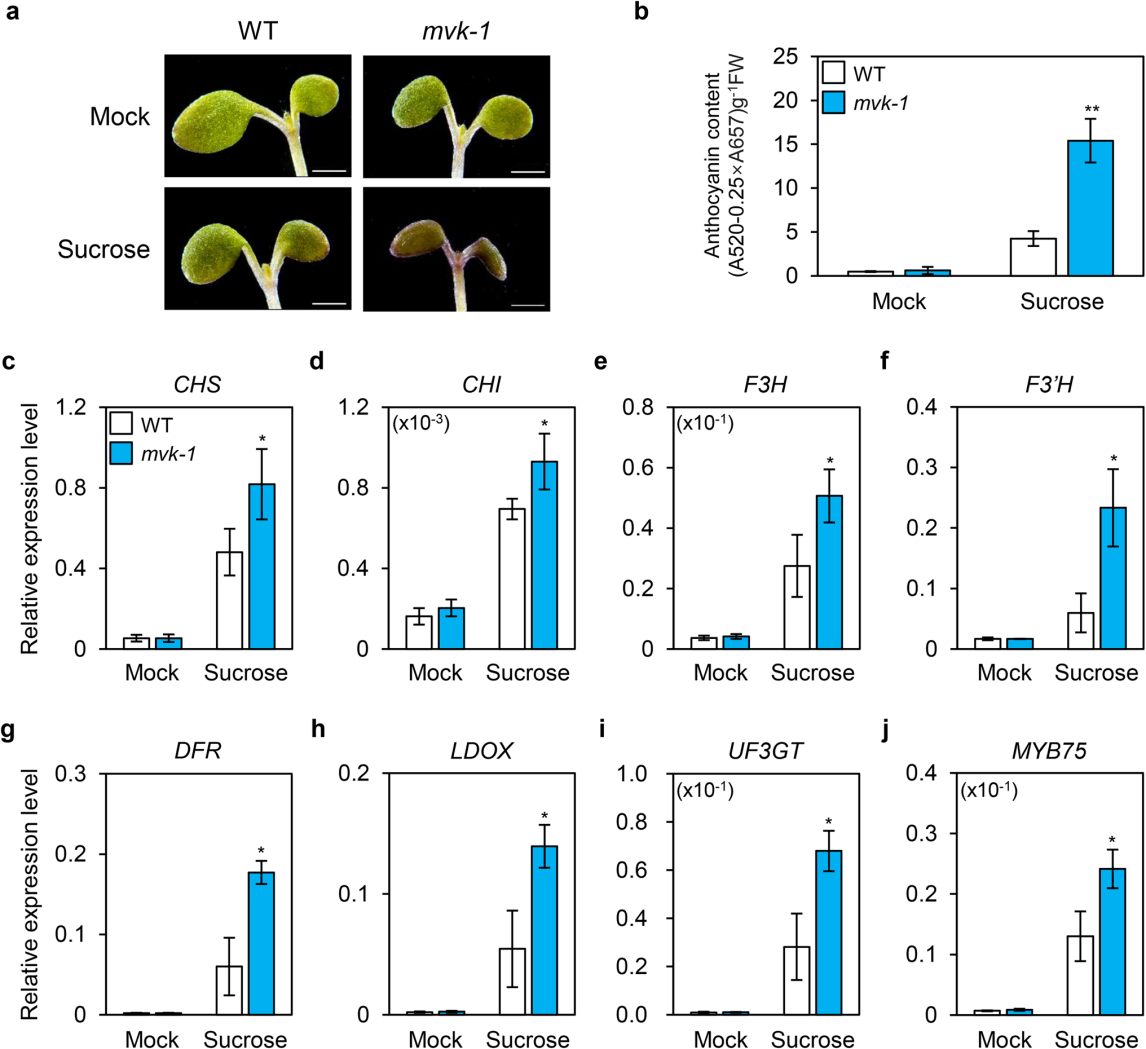
MVK is involved in sucrose-induced anthocyanin biosynthesis. **a** The *mvk-1* mutants accumulate more anthocyanins in response to sucrose treatment. Ten-day-old seedlings of WT and *mvk-1* mutants were grown in half-strength Murashige and Skoog (MS) medium and treated with or without 3% (w/v) sucrose for three days. Scale bar represents 1 mm. **b** Anthocyanin content in WT and *mvk-1* mutants. The anthocyanin content was determined by measuring the absorbance of the plant extracts at 520 nm and subtracting 0.25 times the absorbance at 657 nm. The result was then expressed per gram of fresh weight (FW). The mean and standard deviation (SD) were obtained from three biological replicates. Asterisks indicate significantly different values according to a Student’s *t*-test (*P < 0.05, **P < 0.01). **c**–**j** Expression patterns of anthocyanin biosynthetic and regulatory genes. Relative expression levels of **c** *CHS*, **d** *CHI*, **e** *F3H*, **f** *F3’H*, **g** *DFR*, **h** *LDOX*, **i** *UF3GT*, and **j** *MYB75* in WT and *mvk-1* seedlings. Ten-day-old seedlings grown in half-strength MS medium were treated with or without 3% (w/v) sucrose for three days. White bars represent WT and blue bars represent *mvk-1* mutants. Expression levels were determined by RT-qPCR and normalized to the expression of *UBQ5* reference gene. The mean and SD were obtained from four biological replicates (four independent pooled samples). Asterisks indicate significantly different values according to a Student’s *t*-test (*P < 0.05)

The MVA pathway plays a crucial role in the biosynthesis of a wide range of isoprenoids, including phytohormones (Pulido et al. 2012). It has been reported that CK, GA, and ABA regulate the induction of anthocyanin biosynthesis by sugars in arabidopsis (Loreti et al. 2008). This led us to examine whether mutation of *MVK* affects anthocyanin levels. First, we investigated anthocyanin accumulation in *mvk-1* mutants. To determine the role of *MVK* in anthocyanin accumulation, ten-day-old WT and *mvk-1* plants were grown on half-strength Murashige and Skoog (MS) medium in the absence or presence of 3% sucrose for 3 days. The leaves and shoot apical meristem region of the mutants showed intense purple coloration after being grown on 3% sucrose (Fig. 1a).

Furthermore, a pronounced anthocyanin accumulation phenotype was observed across the abaxial surface of ten-day-old *mvk-1* mutants grown on 5% sucrose medium. (Supplementary Fig. S1). There was no difference in anthocyanin content under mock conditions, whereas *mvk-1* mutants exhibited an almost threefold increase in anthocyanin content compared to WT under 3% sucrose conditions (Fig. 1b).

Next, to identify whether these phenotypes were due to upregulation of anthocyanin biosynthetic genes at the transcriptional level, we examined the relative expression of genes involved in the anthocyanin biosynthetic pathway (*CHS, CHI, F3H, F3’H, DFR, ANS*/*LDOX* and *UF3GT*). Under 3% sucrose conditions, *mvk-1* mutants exhibited higher expression levels of anthocyanin biosynthetic genes compared with WT (Fig. 1c–i). We also measured the relative expression level of *MYB75*, which is a transcription factor composing the MBW complex, involved in the transcriptional activation of anthocyanin biosynthetic genes (Teng et al. 2005). Interestingly, the expression level of *MYB75* was also upregulated in *mvk-1* mutants compared to WT under 3% sucrose conditions (Fig. 1j). Taken together, these findings indicate that a loss-of-function of *MVK* results in enhanced anthocyanin accumulation, likely through transcriptional activation of the MBW complex and the subsequent upregulation of key genes in the anthocyanin biosynthetic pathway.

### High sucrose accumulation in the leaves of *mvk-1* mutants

**Fig. 2:**
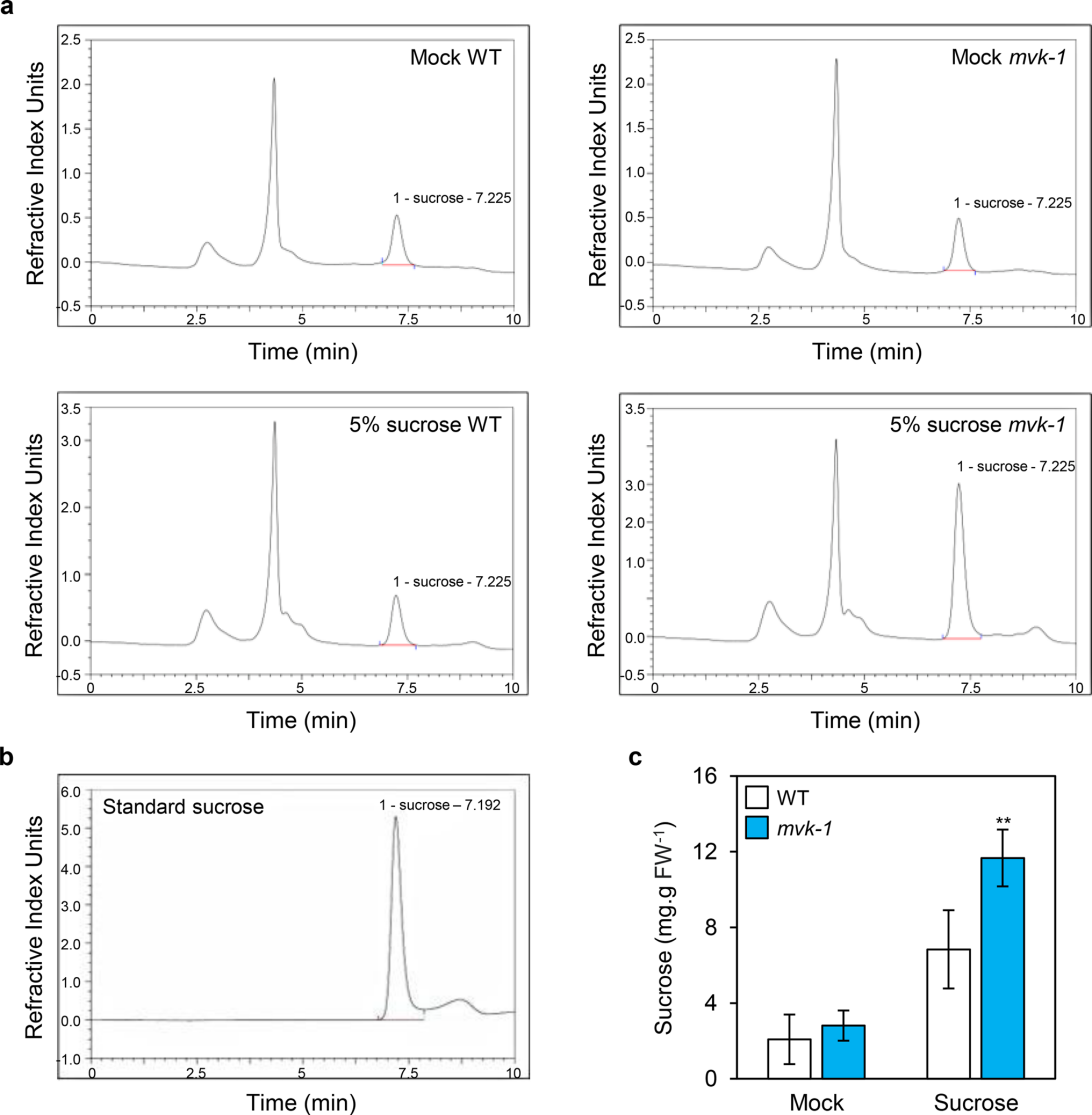
Sucrose content in the leaves of WT and *mvk-1* mutants. **a** Sucrose content in the leaves of ten-day-old WT and *mvk-1* seedlings that were treated with or without 5% (w/v) sucrose for three days. Leaf extracts were analyzed by HPLC. **b** HPLC chromatogram showing the standard sucrose peak. The retention time of sucrose was 7.192 min. **c** Sucrose content in WT and *mvk-1* mutants. White bars represent WT and blue bars represent *mvk-1* mutants. The mean and SD were obtained from more than four biological replicates (four independent pooled samples). Asterisks indicate significantly different values according to a Student’s *t*-test (*P < 0.05, **P < 0.01)

Sucrose is widely recognized as a signaling molecule that induces anthocyanin biosynthesis (Yoon et al. 2021). As shown in Fig. 1, *mvk-1* mutants exhibited increased anthocyanin accumulation under high-sucrose conditions. We hypothesized that this phenotype is associated with elevated sucrose levels. To confirm this hypothesis, we collected leaves from WT and *mvk-1* mutants grown under the same conditions and subjected them to the treatments with or without 5% sucrose. Sucrose content was measured using HPLC. Under mock conditions, no difference was observed in sucrose levels (i.e., peak at ~ 7.2 min) between WT and *mvk-1* mutants. However, *mvk-1* mutants showed higher internal sucrose levels than WT in the presence of 5% sucrose (Fig. 2a, b). The standard sucrose was detected at ~ 7.2 min (Fig. 2b). These results suggest that arabidopsis MVK influences sucrose accumulation, potentially contributing to an enhanced anthocyanin phenotype observed in *mvk-1* mutants.

### Expression patterns of *SUCs* in *mvk-1* mutants

**Fig. 3:**
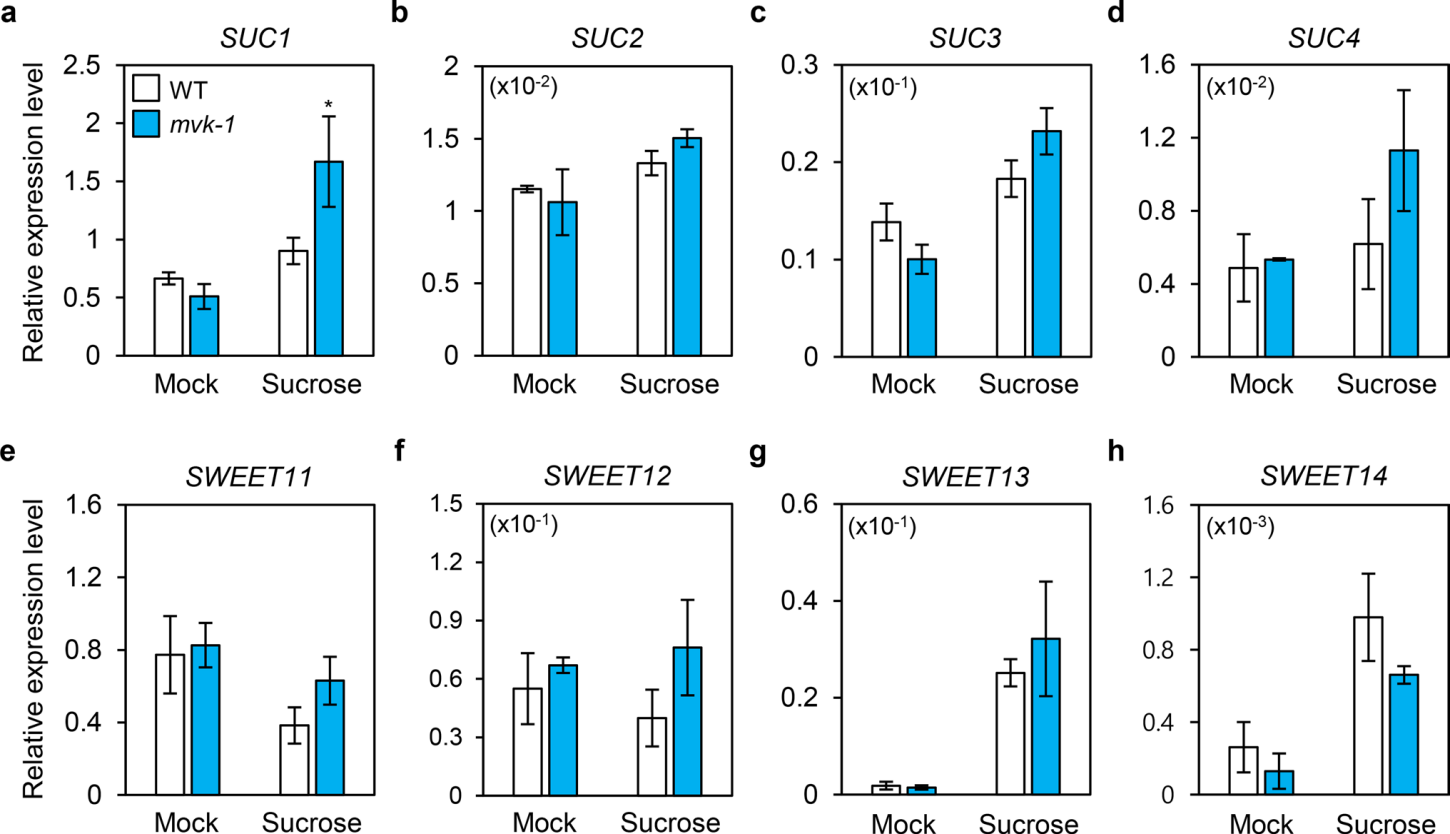
The relative expression levels of sucrose transporter genes in WT and *mvk-1* seedlings. The relative expression levels of **a** *SUC1*, **b** *SUC2*, **c** *SUC3*, **d** *SUC4*, **e** *SWEET11*, **f** *SWEET12*, **g** *SWEET13*, and **h** *SWEET14* in ten-day-old seedlings grown in half-strength MS medium were examined with or without 3% (w/v) sucrose for one day. White bars represent WT and blue bars represent *mvk-1* mutants. Transcript levels were determined by RT-qPCR and normalized to the expression of the *UBQ5* reference gene. The mean and SD were obtained from four biological replicates (four independent pooled samples). Asterisks indicate significantly different values according to a Student’s *t*-test (*P < 0.05)

Sucrose transporters are known to play a central role in regulating sucrose levels in plants (Julius et al. 2017). Since *mvk-1* mutants exhibited increased sucrose accumulation under high exogenous sucrose treatments (Fig. 2c), we investigated whether this phenotype was associated with altered expression of sucrose transporter genes. In order to measure expression of *SUC* genes, we compared their transcript levels between WT and *mvk-1* seedlings under high-sucrose conditions. The expression of *SUC1* was increased in *mvk-1* mutants under high-sucrose conditions (Fig. 3a), whereas that of *SUC2*, *SUC3*, and *SUC4* showed no significant change regardless of sucrose concentrations (Fig. 3b–d).

In addition to *SUC* genes, we also examined the *SWEET* genes. However, no significant differences in the expression of these genes were detected between WT and *mvk-1* mutants under high-sucrose conditions (Fig. 3e–h). Taken together, these results indicated that the mutation of *MVK* specifically alters the expression of *SUC1*, while other sucrose transporters (*SUC2*, *SUC3*, *SUC4*, and *SWEET11-14)* remain unaffected.

### MVK genetically affects SUC1-mediated anthocyanin accumulation

Arabidopsis SUC1 is a plasma membrane-localized sucrose/H⁺ symporter with a distinct expression pattern. SUC1 mediates local sucrose uptake in roots, trichomes, and pollen (Sauer and Stolz 1994; Sivitz et al. 2008; Stadler and Sauer 2019). Since exogenous sucrose strongly induces anthocyanin biosynthesis, and since SUC1 primarily functions in sucrose uptake in roots, we hypothesized that *SUC1* expression is a downstream target of MVK. To investigate this, we generated the CRISPR/Cas9-mediated *suc1-5* mutants in the WT background. The mutants harbor a single-base insertion that results in a premature stop codon in the *SUC1* coding region (Supplementary Fig. S2). Additionally, we generated the *mvk-1 suc1-5* double mutants by introducing the *SUC1* CRISPR/Cas9 construct into the *mvk-1* background to examine their genetic interaction. Sequencing analysis confirmed a single-base insertion in *SUC1* that is identical to the *suc1-5* mutant allele (Supplementary Fig. S2b).

**Fig. 4:**
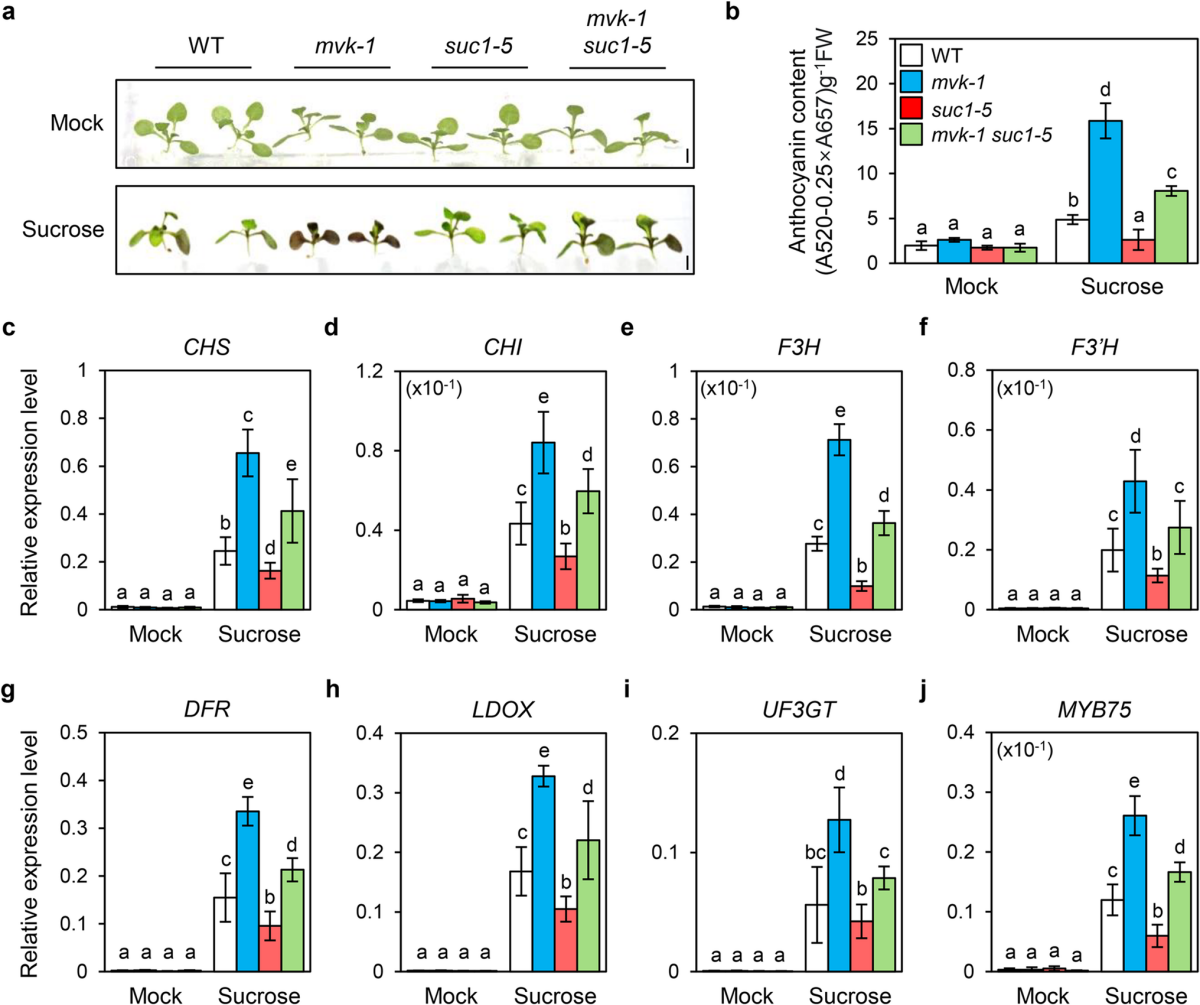
Expression patterns of anthocyanin biosynthetic and regulatory genes **a** Anthocyanin accumulation with or without 3% (w/v) sucrose treatment in WT, *mvk-1*, *suc1-5*, and *mvk-1 suc1-5* mutants. Ten-day-old seedlings of WT, *mvk-1*, *suc1-5*, and *mvk-1 suc1-5* mutants grown in half-strength MS medium were treated with or without 3% (w/v) sucrose for 3 days. The scale bar represents 0.1 cm. **b** Anthocyanin content in WT, *mvk-1*, *suc1-5*, and *mvk-1 suc1-5* mutants. Anthocyanin content was determined by measuring the absorbance of the plant extracts at 520 nm and subtracting 0.25 times the absorbance at 657 nm. The result was then expressed per gram of fresh weight (FW). **c**–**j** The relative expression levels of **c** *CHS*, **d** *CHI*, **e** *F3H*, **f** *F3’H*, **g** *DFR*, **h** *LDOX*, **i** *UF3GT*, and **j** *MYB75* in WT, *mvk-1, suc1-5*, and *mvk-1 suc1-5* seedlings. Ten-day-old whole seedlings grown in half-strength MS medium were treated with or without 3% (w/v) sucrose for three days. The white, blue, red, and green bars represent WT, *mvk-1*, *suc1-5*, and *mvk-1 suc1-5* mutants, respectively*.* Expression levels were determined by RT-qPCR and normalized to the expression of the *UBQ5* reference gene. The mean and SD were obtained from four biological replicates (four independent pooled samples). Different letters indicate significantly different values according to a one-way ANOVA followed by a Duncan’s least significant range test (*P < 0.05)

Consistent with previous studies (Sivitz et al. 2008), *suc1-5* mutants exhibited reduced anthocyanin accumulation under 3% sucrose treatment compared to WT (Fig. 4a, b). Interestingly, *mvk-1 suc1-5* double mutants showed intermediate anthocyanin levels, higher than *suc1-5* mutants but lower than *mvk-1* mutants (Fig. 4a, b). At the transcriptional level, anthocyanin biosynthetic genes were markedly downregulated in *suc1-5* mutants but significantly upregulated in *mvk-1 suc1-5* double mutants compared with WT under sucrose treatment (Fig. 4c-i). Similarly, *MYB75* expression level was reduced in *suc1-5* mutants, whereas it was induced in *mvk-1 suc1-5* double mutants (Fig. 4j). Together, these results suggested that MVK regulates anthocyanin accumulation partially by downregulating *SUC1* expression, while also acting through additional SUC1-independent pathways.

### MVK regulates DELLA stability via GA biosynthesis

**Fig. 5:**
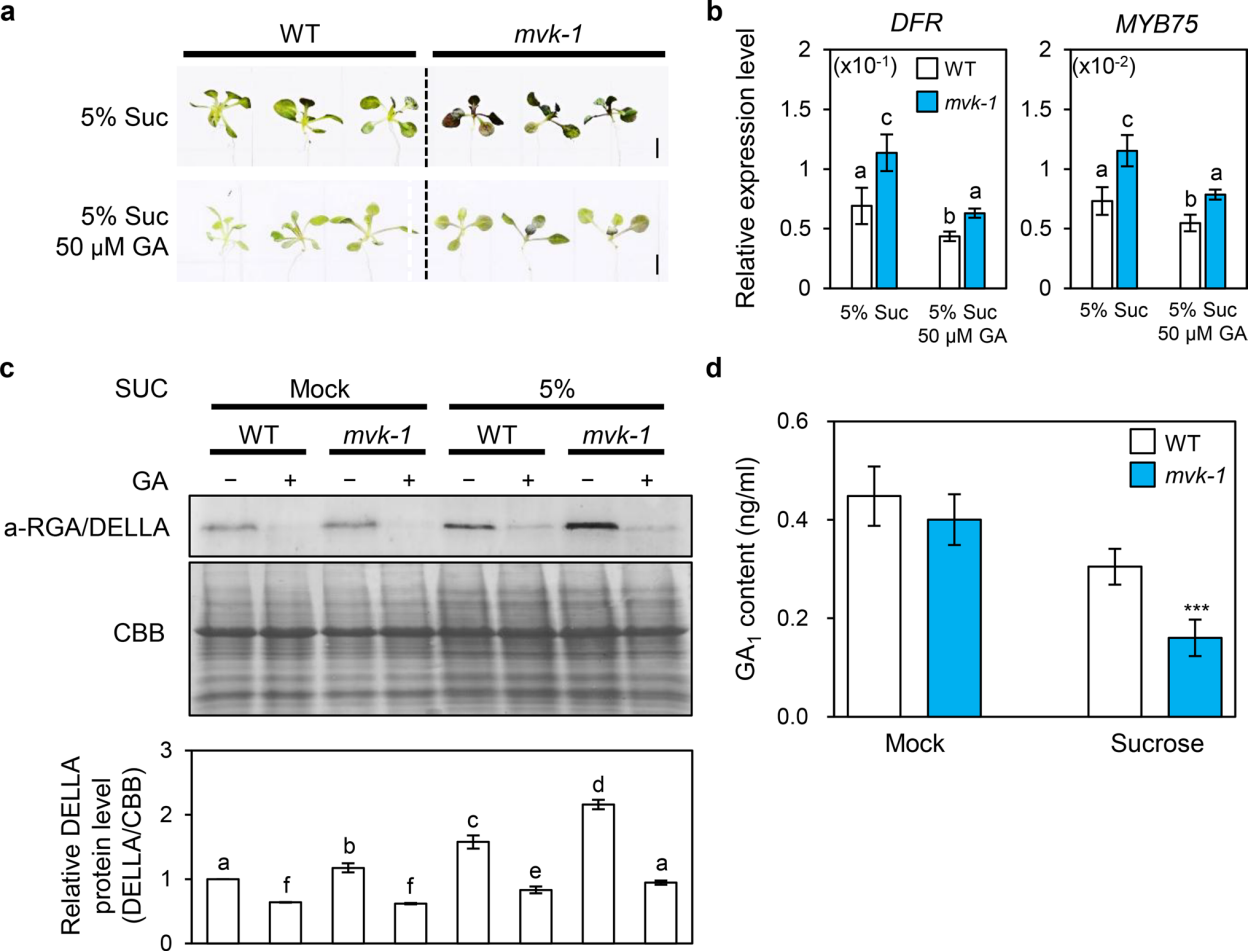
*mvk-1* mutants accumulate lower levels of GA_1_ than WT.** a** Anthocyanin accumulation under 5% (w/v) sucrose with or without 50 μM GA treatments in WT and *mvk-1* mutants. The scale bar represents 0.2 cm. **b** Relative expression levels of *DFR* and *MYB75* under 5% (w/v) sucrose with or without 50 μM GA in ten-day-old WT and *mvk-1* whole seedlings. Transcript levels were determined by RT-qPCR and normalized to the *UBQ5* reference gene. The mean and SD were obtained from four biological replicates (four independent pooled samples). Different letters indicate significantly different values according to a one-way ANOVA followed by a Duncan’s least significant range test (*P < 0.05). **c** Measurement of DELLA protein levels. WT and *mvk-1* mutants were grown under with or without 5% (w/v) sucrose treatments with or without GA. Total DELLA proteins were detected by immunoblotting with an anti-RGA/DELLA antibody. Protein loading was visualized by Coomassie brilliant blue (CBB) staining. DELLA band intensities were quantified using ImageJ and normalized to the corresponding CBB staining signals. Relative DELLA protein levels were calculated by setting the value of WT under 1% sucrose without GA treatment to 1. The mean and SD were obtained from three biological replicates. Different letters indicate significantly different values according to a one-way ANOVA followed by a Duncan’s least significant range test (*P < 0.05). **d** GA_1_ quantification by LC-MS. Ten-day-old whole seedlings of WT and *mvk-1* mutants grown in half-strength MS medium were treated with or without 3% (w/v) sucrose for three days. The mean and SD were obtained from more than four biological replicates (four independent pooled samples). Asterisks indicate significantly different values according to a Student’s *t*-test (***P < 0.001)

Interestingly, *mvk*-*1 suc1-5* double mutants showed significantly higher anthocyanin accumulation compared to *suc1-5* mutants under sucrose treatment (Fig. 4a, b). The MVA and MEP pathway synthesize isoprenoid precursors that are essential for phytohormones, such as brassinosteroids, CK, GA, and ABA (Pulido et al. 2012), and GA suppresses sucrose-induced anthocyanin accumulation (Loreti et al. 2008). Therefore, we hypothesized that MVK may regulate anthocyanin accumulation not only via a SUC1-mediated pathway but also through GA biosynthesis. To confirm this, we compared the anthocyanin accumulation of WT and *mvk-1* mutants under 5% sucrose with or without 50 µM GA treatments. As shown in Fig. 5a, the accumulation of anthocyanins in *mvk-1* mutants under 5% sucrose treatment was attenuated by GA application. Consistent with the anthocyanin accumulation phenotype, the expressions of *DFR* and *MYB75* were significantly downregulated in *mvk-1* mutant but remained higher than WT under 5% sucrose with 50 µM GA treatments (Fig. 5b). Since GA represses sucrose signaling by promoting degradation of DELLA proteins (Li et al. 2014), we measured the level of DELLA proteins in WT and *mvk-1* mutants under sucrose treatment with or without GA treatments. Remarkably, although GA treatment reduced DELLA protein levels in both WT and *mvk-1* mutants, quantification of relative DELLA protein levels normalized to CBB staining showed that DELLA protein abundance remained significantly higher in *mvk-1* mutants than in WT under 5% sucrose conditions, with or without GA treatment (Fig. 5c). Since the mutants exhibited elevated levels of DELLA protein, we investigated whether this was associated with altered GA content. Analysis by LC–MS revealed that GA_1_ levels in the *mvk-1* mutants were significantly lower than those in WT (Fig. 5d).

**Fig. 6:**
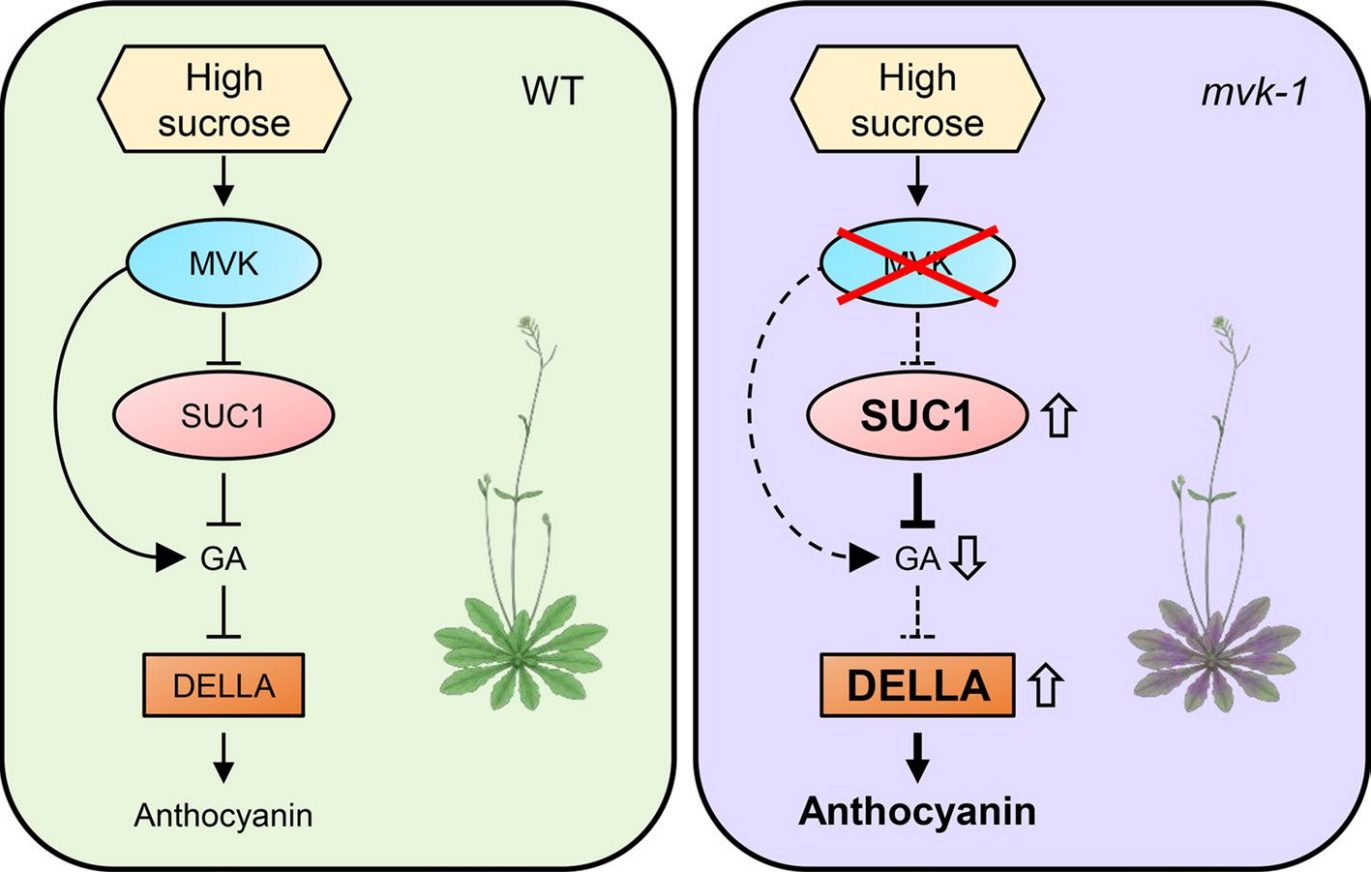
MVK regulates anthocyanin synthesis by modulating *SUC1* expression and GA biosynthesis. The schematic model shows the role of MVK in regulating anthocyanin biosynthesis. MVK represses *SUC1* expression, which in turn downregulates sucrose levels and leads to altered GA levels, both directly and indirectly. Reduced GA levels stabilize the DELLA proteins, thereby activating the MBW complex and enhancing anthocyanin accumulation in arabidopsis

Previous studies have demonstrated that GA biosynthetic genes, such as *Gibberellin 3-oxidase 1* (*GA3ox1*) and *Gibberellin 20-oxidase 1* (*GA20ox1*), exhibit increased expression under GA-deficient conditions, which is consistent with feedback regulatory mechanisms (Fukazawa et al. 2014). Similarly, our results revealed significant upregulation of *GA3ox1* and *GA20ox1* expression in *mvk-1* mutants. In contrast, their expression was downregulation in *suc1-5* mutants compared to WT (Supplementary Fig. S5). Notably, *mvk-1 suc1-5* mutants showed moderate expression levels, which was significantly higher than those of *suc1-5* mutants but lower than those of *mvk-1* mutants. Taken together, our results suggest that reduced GA biosynthesis in *mvk-1* mutants impedes the degradation of DELLA proteins, resulting in higher anthocyanin accumulation than in WT (Fig. 6).

## Discussion

### Loss of MVK enhances the sucrose-specific induction of the anthocyanin biosynthetic pathway

Anthocyanin accumulation is closely linked to sucrose availability, as sucrose transporters, such as SUCs and SWEETs, import extracellular sucrose and thereby induce anthocyanin biosynthesis. Several kinases have been reported to regulate sucrose transporters. For example, Sucrose-Induced Receptor Kinase 1 (SIRK1) phosphorylates and thereby activates several membrane proteins, including SWEET11, under sucrose-specific osmotic response (Wu et al. 2013). Furthermore, Wall-Associated Kinase-Like 8 (WAKL8) phosphorylates SUC2, thereby increasing its transport activity (Xu et al. 2020). However, despite the demonstrated kinase-mediated regulation of sucrose transporters, no studies have reported a mechanism by which such kinase-dependent modulation directly influences anthocyanin biosynthesis.

In this study, we showed that the knockout mutation of *MVK* enhances sucrose-specific anthocyanin accumulation (Fig. 1). *mvk-1* mutants showed increased expression of anthocyanin biosynthetic genes and higher transcript levels of *MYB75*, a key transcriptional regulator of anthocyanin biosynthetic genes (Fig. 1j). Measurement of sucrose contents in leaves revealed that *mvk-1* mutants accumulate higher levels of sucrose under high-sucrose conditions than WT (Fig. 2). Consistently, *mvk-1* mutant plants showed significantly increased *SUC1* expression, indicating that MVK negatively regulates *SUC1* expression. Furthermore, *mvk-1 suc1-5* double mutants showed significantly higher anthocyanin accumulation than the *suc1-5* mutants. Consistent with this, the *suc1-5* mutant exhibited significantly reduced sucrose accumulation compared to WT, and this reduction was restored to significantly higher than WT in *mvk-1 suc1-5* double mutant (Supplementary Fig. S4). This finding suggests that MVK acts as a repressor of *SUC1* and the enhanced sucrose-induced anthocyanin phenotype of *mvk-1* mutants is partially dependent on SUC1.

Taken together, our findings indicate that the enhanced sucrose-induced anthocyanin accumulation in *mvk-1* mutants is primarily driven by SUC1-mediated sucrose uptake. However, the higher anthocyanin levels in *mvk-1 suc1-5* mutants than in *suc1-5* mutants indicate that MVK regulates anthocyanin biosynthesis through an additional regulatory node. This suggests that MVK may coordinate multiple downstream pathways to fine-tune the anthocyanin accumulation.

### The MVA pathway regulates anthocyanin accumulation additionally via GA biosynthesis

Anthocyanin accumulation is tightly controlled by the interplay between sucrose and phytohormones. Auxin and cytokinin promote anthocyanin accumulation through transcriptional activation and antioxidant regulation, whereas ethylene exerts context-dependent effects (Bhaskar et al. 2021; Chandler 2016; Ni et al. 2021). Abscisic acid strongly induces anthocyanin biosynthesis under stress conditions, while GA consistently acts as a negative regulator (Karppinen et al. 2018; Loreti et al. 2008). Moreover, sucrose signaling has been shown to interact with several hormones, including IAA, ABA, MeJA, and SA; however, it is antagonized by GA (Li et al. 2014).

Our results are consistent with the possibility that MVK, a key enzyme in the MVA pathway, influences anthocyanin accumulation in part through altered GA biosynthesis. The *mvk-1 suc1-5* double mutants accumulated more anthocyanin than *suc1-5* mutants (Fig. 4a, b). Furthermore, the double mutants also exhibited reduced root length (Supplementary Fig. S3). These observations suggest the existence of additional regulatory pathway that compensates for the loss of SUC1-mediated sucrose signaling. The dwarfism observed in both *mvk-1* and *mvk-1 suc1-5* mutants (Supplementary Figs. S1, S3) is consistent with the well-established role of the MVA pathway in isoprenoid biosynthesis. This pathway supplies the necessary precursors for GA synthesis (Cho et al. 2022).

In *mvk*-*1* mutants, reduced GA_1_ levels and elevated DELLA protein accumulation (Fig. 5c) alleviate GA-mediated repression of sucrose signaling. This may contribute to enhanced anthocyanin accumulation in *mvk-1 suc1-5* mutants even in the absence of *SUC1* (Fig. 4). Furthermore, exogenous GA treatment attenuated both the hyper-accumulation of anthocyanin and the upregulation of anthocyanin biosynthesis genes in *mvk-1* mutants (Fig. 5a, b). Disruption of MVK function reduces the amount of GGPP-derived GA precursors, consequently stabilizing DELLA proteins (Fig. 5c). Together, these findings suggest that MVK, a component of the MVA pathway, regulates GA biosynthesis, thus adding an additional regulatory module to sucrose-induced anthocyanin accumulation and contributing to the elevated anthocyanin levels observed in *mvk-1 suc1-5* mutants.

While the GA/DELLA pathway may contribute to the SUC1-independent component, it remains unclear whether GA also contributes to *SUC1* upregulation in *mvk-1* mutants. Notably, exogenous GA application did not restore *SUC1* expression in *mvk-1* mutants (Supplementary Fig. S6). This suggests that MVK may regulate SUC1 via a GA-independent pathway. Thus, further studies are needed to elucidate the mechanism by which MVK represses SUC1.

Our study revealed an unrecognized role of MVK in repressing *SUC1* expression (Fig. 3a). In addition, altered GA biosynthesis and DELLA accumulation appear to further modulate this phenotype. Therefore, our findings propose a broader role for the MVK-SUC1 regulatory axis in anthocyanin biosynthesis. Because anthocyanin accumulation is tightly controlled by sucrose availability and stress-induced signaling pathways, the MVK-SUC1 connection may represent a critical node that coordinates primary metabolism, signaling networks, and secondary metabolism. This integration could enable plants to balance growth and stress adaptation by modulating anthocyanin levels.

### Accession numbers

The gene sequences referenced in this article are available in The Arabidopsis Information Resource (https://www.arabidopsis.org/) under the following accession numbers: *MVK*, AT5G27450; *CHS*, AT5G13930; *CHI*, AT3G55120; *F3H*, AT3G51240; *F3’H*, AT3G51240; *DFR*, AT5G42800; *LDOX*, AT4G22880; *UF3GT*, AT5G54060; *MYB75*, AT1G56650; *SUC1*, AT1G71880; *SUC2*, AT1G22710; *SUC3*, AT2G02860; *SUC4*, AT1G09960; *SWEET11*, AT3G48740; *SWEET12*, AT5G23660; *SWEET13*, AT5G50800; *SWEET14*, AT4G25010; *GA3ox1*, AT1G15550; *GA20ox1*, AT4G25420.

## Supplementary Information

Below is the link to the electronic supplementary material.

Supplementary file1 (PDF 724 KB)

## Data Availability

The datasets generated during and/or analyzed during the current study are available from the corresponding author on reasonable request.
