# Supplementary material for: Mevalonate kinase represses anthocyanin biosynthesis via sucrose transporters and gibberellin synthesis pathways in arabidopsis
Source: Plant Cell Rep. 2026 Jun 15;45(7):196. doi: 10.1007/s00299-026-03883-w (PMC13269390; doi:10.1007/s00299-026-03883-w)
Supplement: Supplementary file 1 — Supplementary file1 (PDF 724 KB) [file 299_2026_3883_MOESM1_ESM.pdf]

Supplementary Materials

MEVALONATE KINASE represses anthocyanin biosynthesis via sucrose transporters and gibberellin synthesis pathways in Arabidopsis

Jinku Kang, Sua Cho, Kiyoon Kang, Daewon Kim, Sang-Il Bae, Eunji Shin, So-Yon Park, Gary Stacey, Nam-Chon Paek, and Sung-Hwan Cho

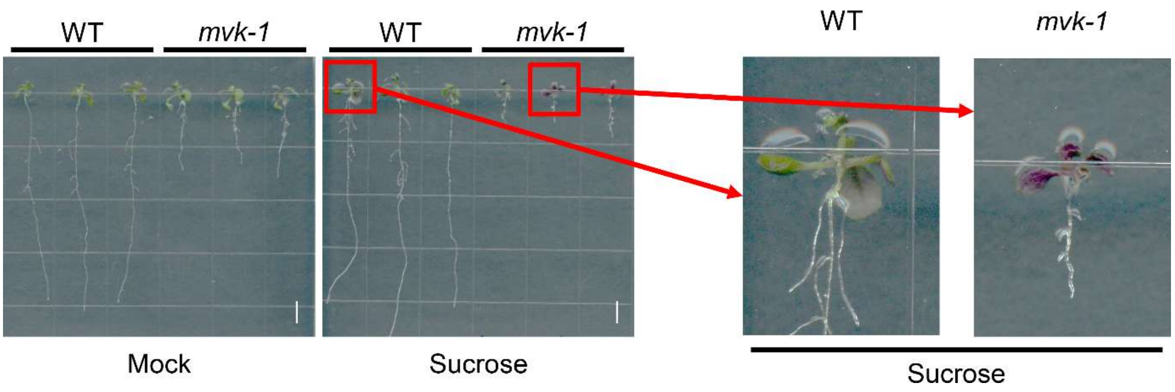

Supplementary Fig. S1 MVK is involved in sucrose-induced anthocyanin biosynthesis. The *mvk-1* mutants accumulate more anthocyanins in response to exogenous sucrose. Ten-day-old seedlings of WT and *mvk-1* mutants grown in half-strength MS medium were treated with or without 5% (v/w) exogenous sucrose for three days. The scale bar represents 1 mm. Red boxes and arrows indicate representative samples of WT (left panel), and *mvk-1* mutant (right panel).

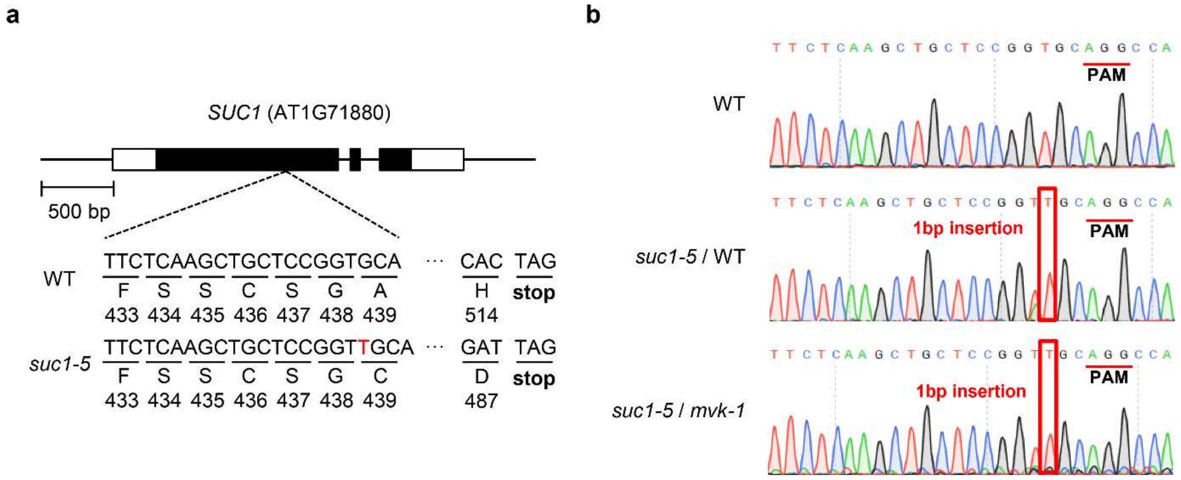

Supplementary Fig. S2 Gene editing information of *suc1-5* and *mvk-1 suc1-5* mutants**.**

a The schematic diagram shows the position of the CRISPR/Cas9 cleavage site, which includes the target sequence and PAM, in *SUC1* gene (AT1G71880). Black and white boxes represent exons and untranslated regions, respectively. Black lines represent introns. Below the diagram, the nucleotides of the CRISPR/Cas9 edited site are compared in WT, *suc1-5*, and *mvk-1 suc1-5* mutants. The insertion of a single adenosine nucleotide (red alphabet) leads to the introduction of a premature stop codon.

b Sanger sequencing results showing a 1-bp insertion in the genomic DNA of *suc1-5* allele compared to WT. The horizontal red lines indicate the PAM sequence, and the red box indicates the predicted double-strand break site.

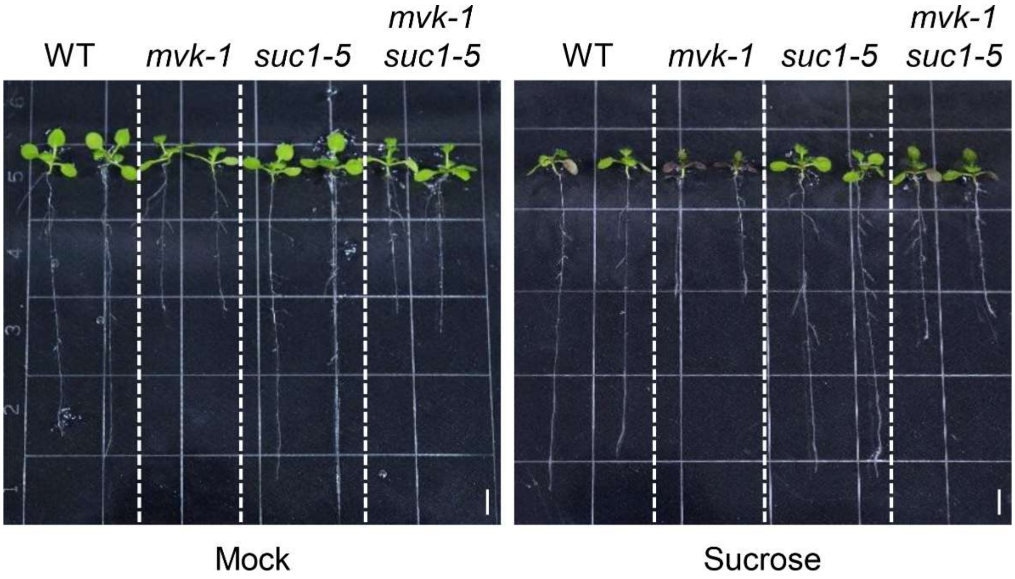

Supplementary Fig. S3 Phenotype of WT, *mvk-1, suc1-5,* and *mvk-1 suc1-5* mutants with or without sucrose treatment.

Ten-day-old seedlings of WT, *mvk-1, suc1-5,* and *mvk-1 suc1-5* mutants were grown in half-strength MS medium and treated with or without 3% (v/w) sucrose for three days. The scale bar represents 0.5 cm.

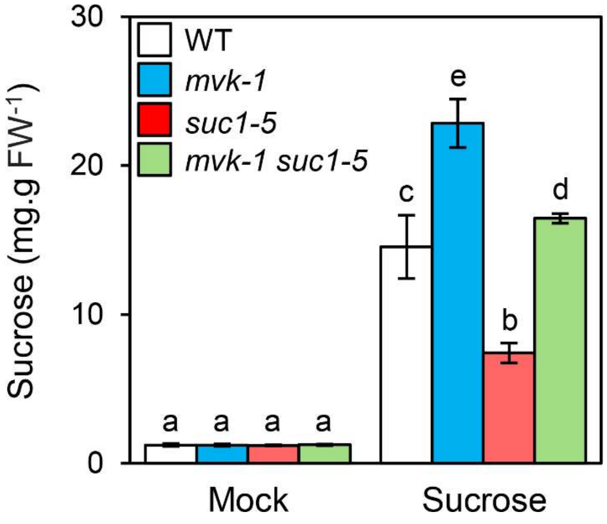

Supplementary Fig. S4 Sucrose content in the leaves of WT, *mvk-1*, *suc1-5*, and *mvk-1 suc1-5* mutants.

The white, blue, red, and green bars represent WT, *mvk-1*, *suc1-5*, and *mvk-1 suc1-5* mutants, respectively. The mean and SD were obtained from four biological replicates (four independent pooled samples). Different letters indicate significantly different values according to a one-way ANOVA followed by a Duncan's least significant range test (**P* < 0.05).

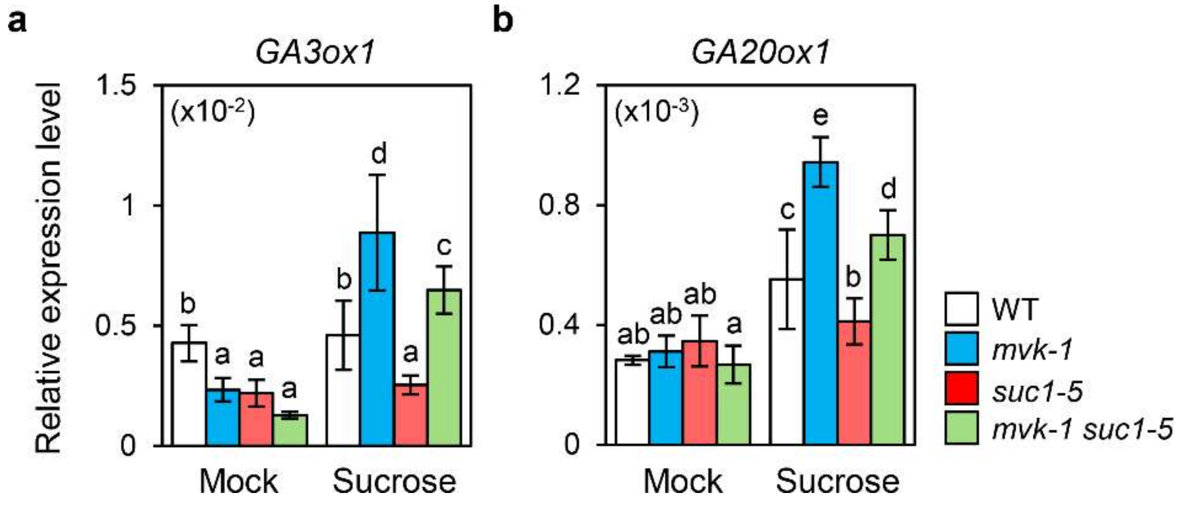

Supplementary Fig. S5 The relative expression levels of GA biosynthesis genes.

The relative expression levels of (**a**) *GA3ox1,* and (**b**) *GA20ox1* in whole seedlings of WT, *mvk-1, suc1-5*, and *mvk-1 suc1-5*. Ten-day-old seedlings grown in half-strength MS medium were treated with or without 3% (w/v) sucrose for three days. The white, blue, red, and green bars represent WT, *mvk-1*, *suc1-5*, and *mvk-1 suc1-5* genotypes, respectively, and are normalized to the *UBQ5* reference gene. The mean and SD were obtained from four biological replicates (four independent pooled samples). Different letters indicate significantly different values according to a one-way ANOVA followed by a Duncan's least significant range test (*P<0.05*).

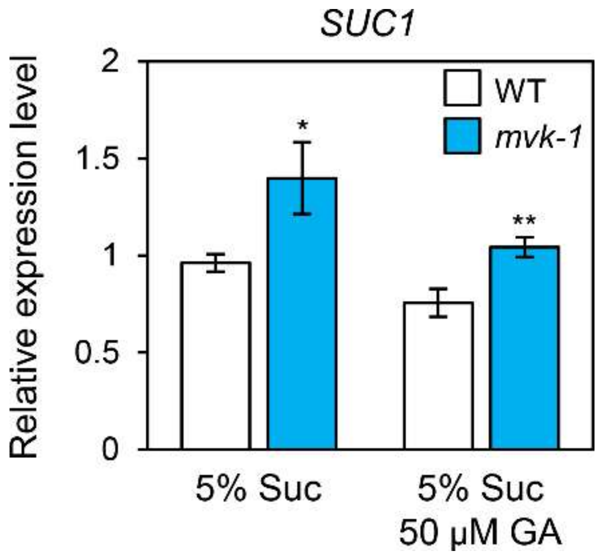

Supplementary Fig. S6 *MVK* regulates *SUC1* through a GA-independent pathway.

The relative expression levels of *SUC1* under 5% (w/v) sucrose with or without 50 μM GA in 10-day-old WT and *mvk-1* whole seedlings. Transcript levels were determined by RT-qPCR and normalized to the *UBQ5* reference gene. The mean and SD were obtained from four biological replicates (four independent pooled samples). Asterisks indicate significantly different values according to a Student's *t*-test (*$P < 0.05$, **$P < 0.01$).

Supplementary Table 1. Primer information in this study.

**A. Primers used for plant transformation**		
Primer name	Forward primer (5'→3')	Reverse primer (5'→3')
pRGEB32-SUC1	TAGGTCTCCCTGCTCCGGTGCGTTTTAGAGCTAGAA	ATGGTCTCAGCAGCTTGAGAATGCACCAGCCGGGAA
**B. Primers used for RT-qPCR**		
Primer name	Forward primer (5'→3')	Reverse primer (5'→3')
qRT-CHS	GGCAAAGAAGCGGCAGTGAAG	CGGAAGGACGGAGACCAAGAAG
qRT-CHI	CCGGTTCATCGATCCTCTTC	ATCCCGGTTTCAGGGATACTATC
qRT-F3H	CAGATCGTTGAGGCTTGTGAGA	GACGAGTCATATCCGCCACTAAGT
qRT-F3'H	GCTCTCGCCGGAGTATTCAA	CCAGCGACGCCTTGTAAATC
qRT-DFR	CTTTGTTCGTGCCACCGTTCG	TCCTTCCTCAGATAAATCAGCCTTCC
qRT-LDOX	TATCAATTTGGCCTAAGACACCAAG	ACCAACTTCTTTCTCTAGACGGTCA
qRT-UF3GT	ATCGAATGAATCGTCAAGCATGAG	TGAGGGATAGAGATGGTGTGGAAAG
qRT-MYB75	AGATAAGAAGAAAGACCAACTAGTG	CCAAGGTGTCCCCCTTTTC
qRT-SUC1	TATTCTCAAGCTGCTCCGGT	TTGCTGCAACGATAAACGCC
qRT-SUC2	GCAGACGGGTGAGTTAGA	GGAGATTGGACCACAGAG
qRT-SUC3	CGGTGGCGGTCAAGGTTTGGC	AGCAGCGAAAGCAGCAACAGACG
qRT-SUC4	CTCTACGCCACGACGACT	ACAACGGGACGAGATGAA
qRT-SWEET11	TCCTTCTCCTAACAACTTATATACCATG	TCCTATAGAACGTTGGCACAGGA
qRT-SWEET12	AAAGCTGATATCTTTCTTACTACTTCGAA	CTTACAAATCCTATAGAACGTTGGCAC
qRT-SWEET13	CGCGTTCTTAGGAGCTGTTC	CCGGGTATGACGGTCGTTAG
qRT-SWEET14	AAACGCTGTGGGATGCTTCA	TTCAAGAGCCCAAGAACCTTCA
qRT-GA3ox1	TCCGAAGGTTTCACCATCACT	TCGCAGTAGTTGAGGTGATGTTG
qRT-GA20ox1	GCCTGTAAGAAGCACGGTTTCT	CTCGTGTATTCATGAGCGTCTGA
